# Comment on ‘Catheter ablation vs. Anti-arrhythmic drug therapy for ventricular tachycardia in ischaemic heart disease: a meta-analysis of randomized controlled trials’

**DOI:** 10.1093/europace/euag077

**Published:** 2026-06-23

**Authors:** Mafalda Griné, Gonçalo Ferraz-Costa, Pedro Sousa

**Affiliations:** Serviço de Cardiologia, Unidade Local de Saúde de Coimbra, Coimbra, Portugal; Faculdade de Medicina da Universidade de Coimbra, Universidade de Coimbra, Coimbra, Portugal; Serviço de Cardiologia, Unidade Local de Saúde de Coimbra, Coimbra, Portugal; Faculdade de Medicina da Universidade de Coimbra, Universidade de Coimbra, Coimbra, Portugal; Serviço de Cardiologia, Unidade Local de Saúde de Coimbra, Coimbra, Portugal

We read with great interest the article by Santoro *et al*., entitled ‘Catheter ablation vs. anti-arrhythmic drug therapy for ventricular tachycardia in ischaemic heart disease: a meta-analysis of randomized controlled trials’.^1^ The authors address a clinically relevant question and provide an updated synthesis of randomized evidence. They report catheter ablation (CA) is associated with a significant reduction in appropriate implantable cardioverter-defibrillator (ICD) therapies, cardiovascular (CV) re-hospitalizations, and adverse events when compared with anti-arrhythmic drug (AAD) therapy, while no significant differences were observed in all-cause or CV mortality. These findings are consistent with current guideline recommendations.^2,3^ However, we would like to highlight several methodological considerations that may help refine the interpretation of this meta-analysis, particularly regarding (i) primary endpoint definition and (ii) trial selection.

First, although the primary endpoint is defined as appropriate ICD therapy (anti-tachycardia pacing [ATP] or shocks), our independent re-checking and extraction of study-level data from the included studies suggest that this outcome is not reported uniformly across trials and is often presented as separate components (ATP and shocks) and/or using different definitions. Moreover, the event counts shown in the forest plots are not readily verifiable as representing the composite endpoint of appropriate therapies (ATP plus shocks) and may, in some cases, reflect shocks-only reporting. We would therefore appreciate clarification as to whether the primary pooled analysis was based on the composite endpoint of appropriate ICD therapy (ATP plus shocks) or on ICD shocks alone. Providing the study-level event table used for data extraction would further enhance transparency. This distinction is clinically relevant, as ATP and shocks are not interchangeable outcomes and may carry different clinical implications.

Second, the inclusion and classification of SMASH-VT and VANISH trials warrant consideration.^4,5^ In Table 1, SMASH-VT is described as comparing ‘ICD plus prophylactic ablation’ with ‘ICD plus AAD’. However, in the original trial, patients undergoing ICD implantation were randomized to prophylactic ablation vs. no ablation (usual care). Consequently, AAD therapy was not mandated in the control arm, and only a minority of patients ultimately received AADs. Therefore, SMASH-VT may not fully represent a randomized comparison between catheter ablation and an AAD-based strategy and may not fully align with the stated PICO framework. In addition, approximately 36% of enrolled patients had ventricular fibrillation, rather than ventricular tachycardia (VT), as the index arrhythmia during invasive electrophysiological testing, which may further limit alignment with the meta-analysis eligibility criteria. The VANISH trial enrolled patients with recurrent VT who were already receiving AAD therapy and randomized them to CA vs. escalation of pharmacological therapy. In this trial design, ablation was performed on top of background AAD therapy, which was generally continued, rather than as direct alternative to drug treatment. Thus, VANISH compares an ablation-based strategy combined with ongoing AAD therapy with a strategy of escalating drug therapy, rather than CA vs. AAD therapy *per se*.

Clarification of endpoint definitions and the rationale for including SMASH-VT and VANISH trials, along with sensitivity analyses restricted to trials directly comparing CA with an active AAD strategy, would strengthen the robustness of the findings and provide more reliable and clinically interpretable data.
